# Alliance of Genome Resources Portal: unified model organism research platform

**DOI:** 10.1093/nar/gkz813

**Published:** 2019-09-25

**Authors:** The Alliance of Genome Resources Consortium, Julie Agapite, Laurent-Philippe Albou, Suzi Aleksander, Joanna Argasinska, Valerio Arnaboldi, Helen Attrill, Susan M Bello, Judith A Blake, Olin Blodgett, Yvonne M Bradford, Carol J Bult, Scott Cain, Brian R Calvi, Seth Carbon, Juancarlos Chan, Wen J Chen, J Michael Cherry, Jaehyoung Cho, Karen R Christie, Madeline A Crosby, Jeff De Pons, Mary E Dolan, Gilberto dos Santos, Barbara Dunn, Nathan Dunn, Anne Eagle, Dustin Ebert, Stacia R Engel, David Fashena, Ken Frazer, Sibyl Gao, Felix Gondwe, Josh Goodman, L Sian Gramates, Christian A Grove, Todd Harris, Marie-Claire Harrison, Douglas G Howe, Kevin L Howe, Sagar Jha, James A Kadin, Thomas C Kaufman, Patrick Kalita, Kalpana Karra, Ranjana Kishore, Stan Laulederkind, Raymond Lee, Kevin A MacPherson, Steven J Marygold, Beverley Matthews, Gillian Millburn, Stuart Miyasato, Sierra Moxon, Hans-Michael Mueller, Christopher Mungall, Anushya Muruganujan, Tremayne Mushayahama, Robert S Nash, Patrick Ng, Michael Paulini, Norbert Perrimon, Christian Pich, Daniela Raciti, Joel E Richardson, Matthew Russell, Susan Russo Gelbart, Leyla Ruzicka, Kevin Schaper, Mary Shimoyama, Matt Simison, Cynthia Smith, David R Shaw, Ajay Shrivatsav, Marek Skrzypek, Jennifer R Smith, Paul W Sternberg, Christopher J Tabone, Paul D Thomas, Jyothi Thota, Sabrina Toro, Monika Tomczuk, Marek Tutaj, Monika Tutaj, Jose-Maria Urbano, Kimberly Van Auken, Ceri E Van Slyke, Shur-Jen Wang, Shuai Weng, Monte Westerfield, Gary Williams, Edith D Wong, Adam Wright, Karen Yook

## Abstract

The Alliance of Genome Resources (Alliance) is a consortium of the major model organism databases and the Gene Ontology that is guided by the vision of facilitating exploration of related genes in human and well-studied model organisms by providing a highly integrated and comprehensive platform that enables researchers to leverage the extensive body of genetic and genomic studies in these organisms. Initiated in 2016, the Alliance is building a central portal (www.alliancegenome.org) for access to data for the primary model organisms along with gene ontology data and human data. All data types represented in the Alliance portal (e.g. genomic data and phenotype descriptions) have common data models and workflows for curation. All data are open and freely available via a variety of mechanisms. Long-term plans for the Alliance project include a focus on coverage of additional model organisms including those without dedicated curation communities, and the inclusion of new data types with a particular focus on providing data and tools for the non-model-organism researcher that support enhanced discovery about human health and disease. Here we review current progress and present immediate plans for this new bioinformatics resource.

## INTRODUCTION AND HISTORY

Model Organism Databases (MODs) and the associated Gene Ontology Consortium (GOC), have been highly successful and are crucial to modern biomedical and biological research (1–7). Increasingly, researchers want information about more than one model organism, especially in the context of human biomedical research, and they would benefit greatly from being able to explore data across many model organisms in a consistent way. To facilitate such cross-organism studies, the Alliance is harmonizing representation of concepts and unifying user interface design for the presentation of data types in common across the GOC and six MODs: the Mouse Genome Database, the Rat Genome Database, the Zebrafish Information Network, FlyBase, WormBase and the *Saccharomyces* Genome Database. These MOD resources extensively curate data for the budding yeast (*Saccharomyces cerevisiae*), worm (*Caenorhabditis elegans*), fruit fly (*Drosophila melanogaster*), zebrafish (*Danio rerio)*, mouse (*Mus* sp.), rat (*Rattus norvegicus)* as well as many closely related taxa. The GOC provides GO annotations for all taxa. To make the case for long-term sustainability, the Alliance is working to make MOD and GOC infrastructure more efficient by sharing computational pipelines and software development.

The Alliance of Genome Resources Portal, reported on here, results from the development and maintenance of a software platform and shared modular infrastructure designed for the coordination of data harmonization activities across the model organism databases. The MODs and GOC remain the focus of organism-specific expert curation of data and are responsible for quality control and submission of data to the Alliance research platform.

## CREATING THE INTEGRATED MODEL ORGANISM PORTAL

The Alliance web portal (www.alliancegenome.org) provides a single point of access to multiple types of genetic and genomic data from diverse model organisms used to study the genetic and genomic basis of human biology, health and disease. The portal is designed with a number of key principles in mind: (a) entity report pages as information ‘hubs’, connecting together a variety of linked data types into single views; (b) a powerful top-level search function, with results faceted by key data types and (c) standardized user-interface components with a common look-and-feel across all organisms. The portal also provides access to large datasets through Application Programming Interfaces (APIs) and data downloads. With each quarterly release of the portal, data from the primary Alliance knowledge centers (as Alliance MODs and GOC, the curation centers, are known) are refreshed, and new data types and displays are added. Version 2.2, released 7/2/2019, includes new extensive representation of gene expression data, inclusion of molecular interaction data, and improved links between orthology, expression and disease data. Release notes can be reviewed here (https://www.alliancegenome.org/release-notes).

## GENE REPORT PAGES

**Figure 1.**
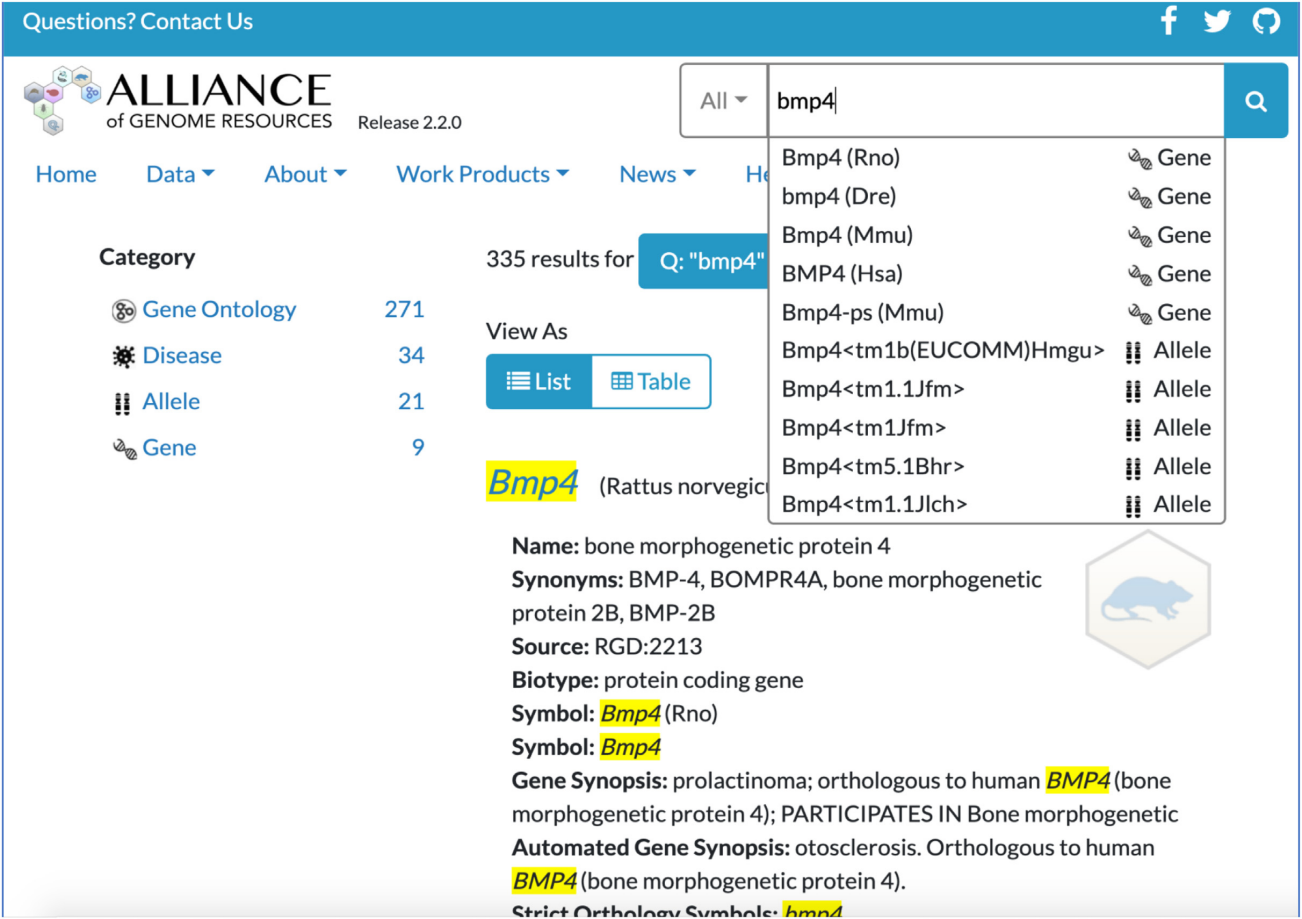
Alliance gene detail page. Here, a quick search box at top right permits search by Gene symbol, GO term, Allele Symbol or Disease term. A drop-down box facilitates term selection. In this example of a search return for *Bmp4*, as noted in the Category listed on the left side, there are 335 results from the search resulting in links to data from 9 Genes, 34 Diseases, 21 Alleles and 271 Gene Ontology terms. Linking to any category extends access to all the underlying data. The page, here presented in List form, can also be viewed as a Table.

Species-specific gene report pages provide detailed information about a gene and its function (Figure 1). They begin with a summary of the gene and include subsections for genomic information, functional (GO) annotations, orthology relationships, phenotypic and disease associations, expression patterns, alleles and molecular interactions. Many details represented include hyperlinks to additional information. Below are descriptions of how elements of this page are generated and refreshed.

### Automated gene descriptions

**Figure 2.**
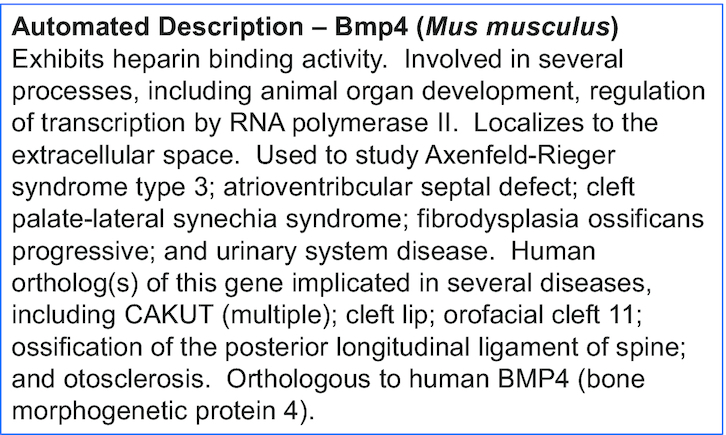
Automated gene description example of mouse gene *Bmp4*.

With so much information available for many genes, intelligent summation of the data is needed to facilitate discoverability and interspecies comparison of gene function. One way of doing this is to provide a short standardized human readable description for every gene, yet manually writing them from an ever-growing literature corpus is a time- and labor-intensive task. To automate this process, we developed an algorithm that summarizes gene function based on curated data. Data include gene associations to Gene Ontology (GO) terms, Disease Ontology (DO) terms, human ortholog data and gene expression data. The problem of generating a text summary from multiple ontology terms annotated to a gene was formulated as an optimization problem. The solution to this problem resulted in a short, template-based paragraph of text containing an optimal combination of ontology terms based on their information content, limited to a predefined maximum text length. The terms in the final description can be either the original annotated term/s or a parent term which groups several of the annotated terms. An example is the automated description provided for mouse gene *Bmp4* which has 291 GO annotations alone (https://www.alliancegenome.org/gene/MGI:88180) (Figure 2). This gene descriptions project has resulted in over 100 000 modular and standardized gene descriptions for purposes of portal display, and can be downloaded from the Alliance.

### Genome features

The Sequence Feature Viewer in the Alliance provides an overview of the genomic structure of genomic features as well as their neighboring elements. Each Alliance model organism group provides a reference genome and gold standard set of genome features within an Alliance-standardized GFF3 format, most often coding and non-coding transcripts that are built into a single standard JBrowse Genome Browser view (8) that allows users to easily browse neighboring genome features easily in each given genome. An overview of the genomic features is shown in individual genome feature pages (e.g. a gene page) using a generic Javascript widget (https://www.npmjs.com/package/genomefeaturecomponent). It utilizes the caching web service provided by the Apollo genome annotation editor (9) to read JBrowse track information.

### Function - Go annotations

**Figure 3.**
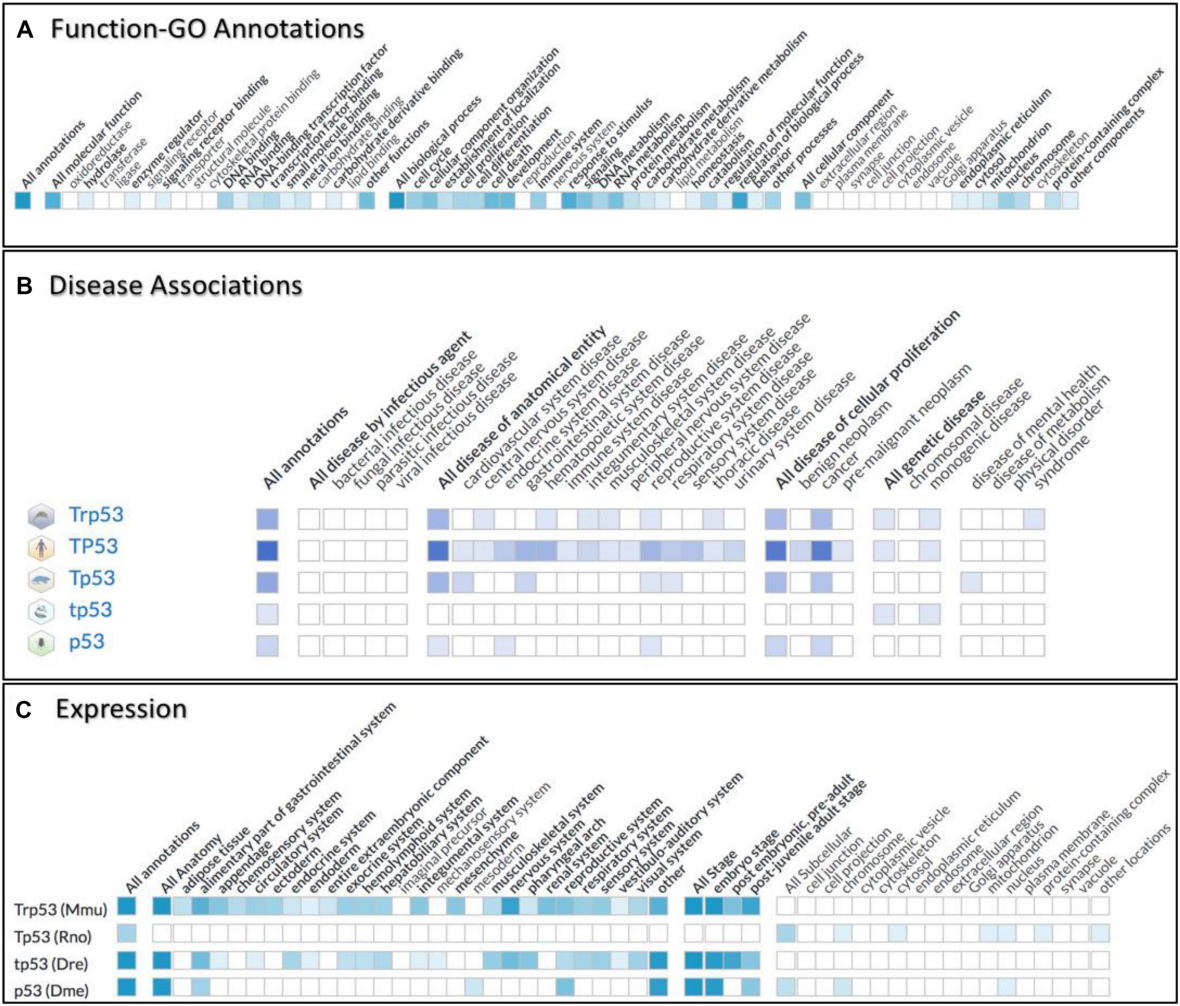
Ribbon summary display for GO biological function, disease associations and expression data. (**A**) Overview of the biological functions of the Trp53 gene, as currently annotated in the Alliance using the Gene Ontology. There are 3 sections informing on the annotated functions, processes and cellular components of Trp53. (**B**) Another high-level view of the mouse Trp53 gene highlighting the currently annotated disease associations using the Disease Ontology. In addition to (A), several orthologs are shown to emphasize interspecies variability. Subsections of the ribbon group related diseases (e.g. ‘All disease of anatomical entity’), with the first column of the group summarizing all annotations for that group. (**C**) A high-level view of expression data of the mouse Trp53 gene and orthologs. The expression ribbon has subsections for anatomical location, developmental stage, and subcellular location. Each ribbon cell represents a high-level anatomical system, tissue, developmental stage, or subcellular component. For each ribbon, the gradient color of each ribbon cell represents the number of currently available annotations for each type of biological data (GO, disease, expression). White cells indicate no data available while color gradient indicate the relative number of annotations available for each cell. Clicking on any colored cell produces a data table with details for the annotations to that section.

Using the controlled vocabulary of the GO, the functions of genes have been carefully annotated over many years by international teams of curators, translating the findings from more than 150 000 published articles into simple, structured and reusable pieces of biological knowledge. A given gene can therefore have tens to hundreds of functions annotated with specific GO terms. The GOC provides a graphical tool, the Ribbon, to summarize those functions and to enable users to drill down to further details as well as to compare the functions of multiple genes easily (Figure 3A). First introduced by the MGD database (10), the GO Ribbon has three sections, reflecting the three branches of the GO ontology, namely Biological Process, Molecular Function and Cellular Component. The high-level terms selected to summarize the functions of genes are carefully designed subsets, also called ‘GO slim’, and darker colors indicate more data (http://geneontology.org/docs/go-subset-guide/). This paradigm is further detailed in *Disease* section below.

One of the challenges of the Alliance consortium was to design a GO slim that would represent the specific experimental findings in all the model organisms yet be general enough to enable useful comparisons between these species, and with human genes. The matrix-like representation of the GO ribbon is a useful way to represent other ontology-based of annotations and thus is also being used to power the Alliance disease and expression ribbons (Figure 3B, C). The REACT component is freely available as an NPM package (https://www.npmjs.com/package/@geneontology/ribbon) and a more generic ribbon that can easily be integrated in any website (independent of the framework used) is in progress. Community feedback and requests are also welcome and can be submitted at the public GitHub repository (https://github.com/geneontology/ribbon).

### Orthology

**Figure 4.**
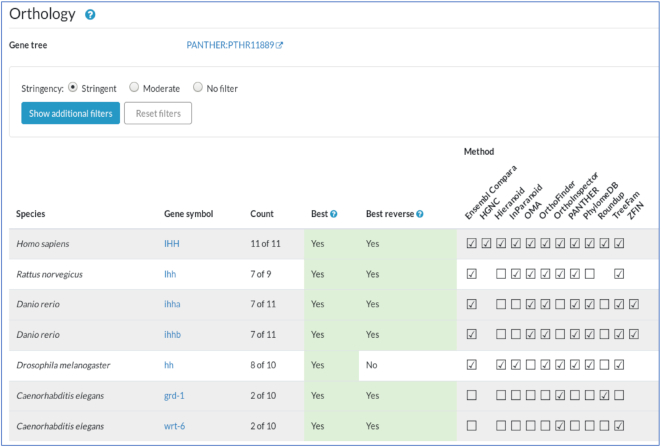
Orthology section in the gene report. Mouse *Ihh* gene report shown. Data are presented in a table based on an integrated representation of orthology inferences by multiple algorithms, listed in ‘Method’ columns. The ‘Count’ indicates the number of methods that call a specific ortholog pair; ‘Best’ and ‘Best reverse’ are based on the counts. Multiple filtering options are offered, including several precomputed options labelled ‘Stringent’ and ‘Moderate’. The ‘Stringent’ option includes all orthologs called by three or more methods that are also a best count OR a best reverse count; an ortholog called by two methods is included if it is both a best count AND a best reverse count; an ortholog predicted by ZFIN or HGNC is always included, regardless of count. Additional information about filtering can be found in the help button pop-up. At the top of the Orthology section is a link to a customized Panther ‘Gene tree’, which incorporates a phylogenetic view and options such as multiple sequence alignments.

The representation of orthology is essential to comparative genomic studies and to interpretation and inferential conclusions of model organism studies. One of the key initial goals of the Alliance is to generate, display and provide a common set of orthologs for all the organisms represented in the Alliance, including human; this common set is used for all cross-species data comparisons and tables. In the Orthology section within each gene report, multiple interactive presentations of orthology data are provided. Ortholog inferences from methods benchmarked by the Quest for Orthologs Consortium (11), as well as manually curated ortholog inferences from HGNC and ZFIN, have been incorporated into a tabulated presentation (Figure 4).The ortholog inferences from the different methods have been integrated using the DRSC Integrative Ortholog Prediction Tool (DIOPT) (12). DIOPT assigns a score/count based on the number of methods that call a specific ortholog pair. The DIOPT approach allows flexibility in the choice of algorithms and in the level of stringency applied; in the Orthology table these parameters can be specified. Three precomputed stringency options, described in the filtering help pop-up, are provided. The most stringent of these options is used as the default ortholog set for this table and for other cross-species data comparisons within the Alliance. Viewing ortholog inferences in the context of a phylogenetic tree is also an option, by following the link to the PANTHER Tree Viewer (13). A customized PANTHER view, developed for the Alliance, shows a ‘trimmed’ tree that includes the Alliance species plus one or two outgroups to each (e.g. http://pantherdb.org/treeViewer/treeViewer.jsp?book=PTHR11889&species=agr). The Alliance orthology data are updated approximately yearly, reflecting changes in the underlying gene models, improvements in the orthology assessment methods, and the addition of new orthology methods. These data can be accessed via several Alliance APIs (see details below).

### Phenotypes

Currently, phenotype data tables presented on each Alliance gene page show information associated with genes and alleles. Data include the species-specific phenotype ontology term, the associated genetic entity with links back to the specific MOD/knowledge center website for alleles and citation data. Data can be sorted in page by any of these values and downloaded as a tab-delimited file. Planned future improvements include more harmonization of the ontologies, implementation of a phenotype summary ribbon, and the addition of pages for specific alleles and genotypes/strains etc. to display more complex models and transgenics. An important challenge is the reconciliation of phenotype terminologies and curation workflows across the Alliance groups. Work to develop shared phenotype classifications and representations across the Alliance data is on-going and includes collaboration with other phenotype ontology integration efforts such as the Monarch Initiative (14).

### Disease associations

Each Alliance gene page has a Disease Associations section that shows a summary ribbon of the disease associations for the gene (Figure 3B). Subsections of the ribbon group related diseases (e.g. ‘All disease of anatomical entity’), with the first column of the group summarizing all annotations for that group. Individual ribbon boxes are high level terms from the Disease Ontology (DO) (15). As with the Function and Expression ribbons, these are shaded when annotations are present, with darker hues indicating more data. Clicking on any shaded box will insert a table showing the individual disease annotations that contribute to that box, with data columns similar to the disease pages (described below). Links to the annotated data at each MOD resource are provided within the table. The ‘Compare to Orthologous Gene’ section allows users to view disease annotations for all or selected orthologs from other species included within the Alliance. By default, only the orthologs that are most likely (‘more stringent’) are used, but a set of orthologs of lower confidence can be selected (‘less stringent’). When orthologs are selected, a new ribbon row appears and the annotation table is updated, facilitating comparison between species.

In addition to the gene page, each Alliance disease page shows a DO term, its synonyms, cross references to other disease resources, parent/child terms within the DO and annotation sources. The Associations table lists all models of that disease (or its subclasses) for the species included within the Alliance. Annotation information includes gene, species, annotated genetic entity used, the association type, evidence, source and citations. Both direct annotations and annotations inferred by orthology are included. Genetic entities include gene and allele, with more complex genomic models, such as genotypes, fish, strains and defined populations to be added soon. The ‘association type’ describes the nature of the relationship between the given genetic entity and the disease, such as ‘is implicated in’, ‘implicated via orthology’ or ‘biomarker of’. Data in the table may be filtered or sorted by various parameters, and can be downloaded as a tab-delimited file. (The entire Alliance disease annotation set may be downloaded from the ‘Data’ link at the top of any page.)

### Expression

Gene expression data in the Alliance currently include integrated wild type expression. To date, the core metadata aspects, such as where and when of gene expression, have been captured using relevant bio-ontologies. Each Alliance gene page includes a section for gene expression data initially showing a summary ribbon of the wild-type expression data for the gene (Figure 3C). The ribbon is broken into subsections for anatomical location, developmental stage, and subcellular location. Each ribbon column represents a high-level anatomical system, tissue, developmental stage, or subcellular component. Anatomical structures and developmental stages used in the ribbon are UBERON (16) terms up to which the model organism anatomy and stage ontology terms can be mapped. The cellular component ribbon terms are high level GO Cellular Component terms. The same ribbon terms are used for all species. Boxes in the ribbon are filled in when annotations exist. The intensity of the color in each box is indicative of the number of expression annotations that are represented in that ribbon box; the darker the color, the more annotations there are. White cells have no annotations. Clicking on colored cells produces a data table with details for the relevant annotations. Links to the original data at the MOD and the original source publication are available in the data table. The ortholog picker, located above the expression summary ribbon provides a means to add orthologs of the gene to the ribbon summary. Only orthologs having expression annotations are provided for selection. Once a ribbon column is clicked, and a data table has been shown, the set of data in the data table may be downloaded by clicking the ‘Download’ button below the table.

### Alleles and variants

Endogenous alleles and their phenotypes are summarized in a table on the gene page, allowing efficient comparison of the alleles described for a particular gene. Phenotypes are described using species-specific phenotype ontology terms (see ‘Phenotypes’ section, above). Also included, if relevant, are synonyms, associated diseases for alleles used to model human disease, and variant information for alleles mapped to the genome.

Variants for phenotypic alleles are represented using Human Genome Variation Society (HGVS) notation standards. Basic information about each variant, including variant type (e.g. point mutation, deletion) and molecular consequence (e.g. missense, stop gained, frameshift) is displayed and linked to the Alliance JBrowse Alleles/Variants track. Variant information at the genomic level is submitted from the MODs to the Alliance in a format that allows the creation of common variants files (such as VCF files) and HGVS names. These are created at the Alliance and will be available for download/API.

### Molecular interactions

Molecular interactions (e.g. protein–protein and protein–DNA interactions) between genes and gene products are now available on gene pages for genes from all seven Alliance species including humans. These interactions are sourced from two WormBase and FlyBase as well as two external interaction databases, BioGRID (https://thebiogrid.org/) (17) and the IMEx consortium (http://www.imexconsortium.org/) (18). The entire dataset can be downloaded from the Alliance Downloads page in tab-delimited PSI-MITAB 2.7 format). The gene page Molecular Interactions section displays a table of molecular interactions for the gene of focus, providing the identity of the gene for the interactor, the interactor molecule types (e.g. protein, DNA, RNA) for each interactor, interaction detection methods, source identifiers (with hyperlinks) and references as PubMed IDs (with hyperlinks). Preset sort options are provided and individual columns can be filtered for convenience. All data presented in the table can be downloaded in tab-delimited format via the ‘Download’ button below the table.

## PORTAL SEARCH

A search box at the top right of every Alliance web page provides an entry point into Alliance data. The search tool returns the broadest possible set of results (to limit search dead ends), sorts them based on a relevance score, and offers filters for further refinement of results. Upon entering text into the search box, autocomplete suggestions offer direct links to specific Alliance pages. For example, typing in ‘axon g’ returns suggestions for the GO term ‘axon guidance’ and the disease term ‘giant axonal neuropathy 1’, among others. If an autocomplete suggestion is not chosen, a list of search results is returned, with the most relevant results at the top of the list. Results are scored based on the number of query terms matched, the type of object attribute matched, and whether the match is partial or exact. For example, an exact match to a gene's official symbol is scored more highly than an exact or partial match to one of that gene's synonyms. A succinct summary is given for each result, with information on how it matched the query term(s), providing the user a means of assessing the quality of each result quickly.

Importantly, the search tool has been imbued with additional ‘knowledge’ about key words and relationships between objects. Common names for organisms are recognized, such that the query term ‘fish’ promotes results for zebrafish objects. Matches to a gene's orthologs are recognized, such that a search for ‘SMAD6’ returns not only the mammalian genes that go by that name, but also the fly ‘Dad’ and nematode ‘tag-68’ genes. Relationships between ontology terms are also built into the search, such that a search for ‘cell-cell junction’ returns matches to the more specific term ‘gap junction’, or a search for ‘eye’ returns matches to ‘retina’, etc.

A key design goal of our search feature is the presentation of results as *facets*. These provide the user with categories/subcategories allowing them to narrow their search results. For example, a list of genes can be filtered for intersection with specific disease, GO, anatomy or biotype terms. The facets also provide counts of entities within that category. These numbers change after a facet is selected by the user to reflect the remaining results available. This provides the user a means to navigate through the data available at the Alliance.

## IMPLEMENTATION AND ARCHITECTURE

### Standardization of data

Our overall goal of standardizing data across model organisms requires developing a common data vocabulary and schema. An important example of this is the use of biomedical ontologies - standardizing on which ontologies to use (e.g. the Alliance-wide adoption of the Disease Ontology for human disease associations), and defining principles for how they should be applied. Unifying use of ontologies across the Alliance groups significantly reduces the complexity of the data, improves reusability, and allows queries to be performed across species.

### Software and hardware infrastructure

Since beginning the implementation of the Portal, we have been iteratively developing an architecture and a system for continuously integrating new functionality and automatically deploying the functionality as it is developed. All code produced within the Alliance is Open Source and can be found in GitHub (https://github.com/alliance-genome/). To reduce the complexity of the project, we chose to use three widely used languages for which considerable expertise exists amongst the Alliance developers: JavaScript, Java and Python. Each repository has a Docker (https://www.docker.com/) file which can be used for deploying the software within a container. We use GoCD (https://www.gocd.org/) to implement Continuous Integration/Continuous Deployment (CI/CD); that is, when changes are pushed to the ‘master’ branch of our repository, pipelines automatically rebuild and deploy the software. These pipelines are especially important to make sure we are able to deploy new functionality quickly thus increasing the rate at which we can produce new functionality for the project.

The Alliance Portal is designed to be deployed in a cloud environment. To date, we have used Amazon Web Services (AWS). The Alliance involves developers across multiple institutions on two continents, and cloud-based development has significantly lowered barriers to participation.

### Database infrastructure

The Alliance portal is backed by two complementary databases: a main datastore, which acts as a hub for the aggregation of data; and a supplementary database to drive our faceted search service. For our datastore, we currently use the graph database platform Neo4j (www.neo4j.com), as it provides a natural way to model and store and query genetic and genomic entities, annotations on them, and the relationships between them. For our search service, we use the widely-used ElasticSearch (www.elastic.co). Database technology is an evolving field, however, and we periodically reassess our technology choices to ensure that they scale to our modelling and service requirements.

### Other architecture components

These include a File Management System for keeping track of data submitted by the MODs, software for importing data into the datastore, and several tools for retrieving data out of the datastore. These tools include a file generator for making files including VCF, Orthology, DAF files; a REST API for providing data to the web interface; and an indexer for creating the search index.

### Quality control

There are three main ways that ensure we consistently produce a high quality product for our users: software tests, curator testing, and user feedback. For software, we have both unit tests and integration tests; over time we will continue to add more tests as we gain more data and data types. In addition to software tests, curators test all software releases before they are installed on the website. Any issues curators find are addressed by developers before release, including adding new software tests, as appropriate. Once the software is released to the public we track user helpdesk requests and make changes when applicable.

### Releases

Alliance versions are tracked with regular software releases every quarter on average. Release notes summarize updates and new data details (https://www.alliancegenome.org/release-notes).

## COMMUNITY OUTREACH AND PUBLIC ACCESS

### Web site

The URL for the Alliance website is www.alliancegenome.org. The Alliance web portal first public release was in October 2017. Early functionality included ability to search by gene, disease, or GO term and included orthology, gene detail pages, and extensive link-outs to contributing bioinformatics resources. With each release, new data and functionality are added.

### API

Found at https://www.alliancegenome.org/api/swagger-ui/ is the Alliance genome RESTful API for access to Alliance data. This link can be found under ‘Data’ at the top of each web page.

### Help and documentation

Immediate contact to the Alliance can be made through the HelpDesk at help@alliancegenome.org. Details for contacting Alliance for help, and documentation of details about data sources and presentation in the Alliance site are found at https://www.alliancegenome.org/help. Helpful FAQs can be found at https://www.alliancegenome.org/faq.

### Social media

The Alliance maintains an active and growing Twitter account (@alliancegenome) and FaceBook page. @alliancegenome publicizes new and updated features with ‘Tweetorials’, each comprised of a short series of tweets illustrating the details of a feature. @alliancegenome also tweets news and items of interest from the Genetics Society of America, the MODs and other useful sources.

## FUTURE DIRECTIONS

The future of the Alliance Portal entails several components including (i) the continued addition of new data types into the Alliance, (ii) the addition of data from other model organisms and (iii) enhancing the ability to navigate between genomes and unlock the power of comparative genomics. A few immediate extensions will include representation of genome variants, improvements in pathway analysis, and improvements in representation of alleles and phenotypes. Initial inclusion of additional organisms will focus on those that have a well-curated community databases such as for *Schizosacchoromyces pombe* PomBase (https://www.pombase.org/) and *Xenopus laevis* (XenBase, http://www.xenbase.org).

An upcoming major extension will be the inclusion of variant data. The variants information at the genomic level is currently available at the Alliance. We are planning to use this information to predict the molecular consequences on known transcripts (using tools like the Variant Effect Predictor) and proteins. These predicted results will be displayed in JBrowse and in the genome viewer, including filtering options. Variants with relation to protein domains, and amino acid conservation will also be available, including options for cross-species comparison. We will also expand the variant information to variants not associated to alleles, for example variants identified by high throughput methods (e.g. SNPs), and variants in non-protein coding regions. The ultimate goal is to allow cross-species query of identical or related variants (using gene name, variants name, or genomic coordinates) and their associated phenotypes and/or diseases.

## CITING THE ALLIANCE

For a general citation of the Alliance of Genome Resources, researchers should cite this article. To cite a specific entry or dataset, use the following format: [Type of] data were retrieved from the Alliance of Genome Resources, URL: https://www.alliancegenome.org; [the date you retrieved the data cited and Alliance release (e.g. 2.2.0, found in the header of every Alliance page)]

## APPENDIX

(alphabetical) Julie Agapite, Laurent-Philippe Albou, Suzi Aleksander, Joanna Argasinska, Valerio Arnaboldi, Helen Attrill, Susan M Bello, Judith A. Blake, Olin Blodgett, Yvonne M. Bradford, Carol J. Bult, Scott Cain, Brian R. Calvi, Seth Carbon, Juancarlos Chan, Wen J. Chen, J. Michael Cherry, Jaehyoung Cho, Karen R Christie, Madeline A. Crosby, Jeff De Pons, Mary E Dolan, Gilberto dos Santos, Barbara Dunn, Nathan Dunn, Anne Eagle, Dustin Ebert, Stacia R. Engel, David Fashena, Ken Frazer, Sibyl Gao, Felix Gondwe, Josh Goodman, L. Sian Gramates, Christian A. Grove, Todd Harris, Marie-Claire Harrison, Douglas G. Howe, Kevin L. Howe, Sagar Jha, James A. Kadin, Thomas C Kaufman, Patrick Kalita, Kalpana Karra, Ranjana Kishore, Stan Laulederkind, Raymond Lee, Kevin A. MacPherson, Steven J. Marygold, Beverley Matthews, Gillian Millburn, Stuart Miyasato, Sierra Moxon, Hans-Michael Mueller, Christopher Mungall, Anushya Muruganujan, Tremayne Mushayahama, Robert S. Nash, Patrick Ng, Michael Paulini, Norbert Perrimon, Christian Pich, Daniela Raciti, Joel E. Richardson, Matthew Russell, Susan Russo Gelbart, Leyla Ruzicka, Kevin Schaper, Mary Shimoyama, Matt Simison, Cynthia Smith, David R. Shaw, Ajay Shrivatsav, Marek Skrzypek, Jennifer R. Smith, Paul W. Sternberg, Christopher J. Tabone, Paul D. Thomas, Jyothi Thota, Sabrina Toro, Monika Tomczuk, Marek Tutaj, Monika Tutaj, Jose-Maria Urbano, Kimberly Van Auken, Ceri E. Van Slyke, Shur-Jen Wang, Shuai Weng, Monte Westerfield, Gary Williams, Edith D. Wong, Adam Wright, Karen Yook
